# Whole-Blood Mitochondrial DNA Copies Are Associated With the Prognosis of Acute Respiratory Distress Syndrome After Sepsis

**DOI:** 10.3389/fimmu.2021.737369

**Published:** 2021-09-07

**Authors:** Tamara Hernández-Beeftink, Beatriz Guillen-Guio, Héctor Rodríguez-Pérez, Itahisa Marcelino-Rodríguez, Jose M. Lorenzo-Salazar, Almudena Corrales, Miryam Prieto-González, Aurelio Rodríguez-Pérez, Demetrio Carriedo, Jesús Blanco, Alfonso Ambrós, Elena González-Higueras, Nancy G. Casanova, Manuel González-Garay, Elena Espinosa, Arturo Muriel, David Domínguez, Abelardo García de Lorenzo, José M. Añón, Marina Soro, Javier Belda, Joe G. N. Garcia, Jesús Villar, Carlos Flores

**Affiliations:** ^1^Research Unit, Hospital Universitario N.S. de Candelaria, Universidad de La Laguna, Santa Cruz de Tenerife, Spain; ^2^Research Unit, Hospital Universitario Dr. Negrin, Las Palmas de Gran Canaria, Spain; ^3^Genomics Division, Instituto Tecnológico y de Energías Renovables (ITER), Tenerife, Spain; ^4^CIBER de Enfermedades Respiratorias, Instituto de Salud Carlos III, Madrid, Spain; ^5^Intensive Care Unit, Complejo Asistencial Universitario de Palencia, Palencia, Spain; ^6^Department of Anesthesiology, Hospital Universitario de Gran Canaria Dr. Negrín, Las Palmas de Gran Canaria, Spain; ^7^Department of Medical and Surgical Sciences, University of Las Palmas de Gran Canaria, Gran Canaria, Spain; ^8^Intensive Care Unit, Complejo Hospitalario Universitario de León, León, Spain; ^9^Intensive Care Unit, Hospital Universitario Rio Hortega, Valladolid, Spain; ^10^Intensive Care Unit, Hospital General de Ciudad Real, Ciudad Real, Spain; ^11^Intensive Care Unit, Hospital Virgen de la Luz, Cuenca, Spain; ^12^Department of Medicine, The University of Arizona, Tucson, AZ, United States; ^13^SPECTRUM, LLC, Phoenix, AZ, United States; ^14^Department of Anesthesiology, Hospital Universitario N.S. de Candelaria, Santa Cruz de Tenerife, Spain; ^15^Intensive Care Unit, Hospital Universitario La Paz, IdiPAZ, Madrid, Spain; ^16^Anesthesiology and Critical Care Department, Hospital Clinico Universitario of Valencia, Valencia, Spain

**Keywords:** ARDS, mitochondria, DAMPs, whole blood, mtDNA, survival

## Abstract

Acute respiratory distress syndrome (ARDS) is an inflammatory process of the lungs that develops primarily in response to pulmonary or systemic sepsis, resulting in a disproportionate death toll in intensive care units (ICUs). Given its role as a critical activator of the inflammatory and innate immune responses, previous studies have reported that an increase of circulating cell-free mitochondrial DNA (mtDNA) is a biomarker for fatal outcome in the ICU. Here we analyzed the association of whole-blood mtDNA (wb-mtDNA) copies with 28-day survival from sepsis and sepsis-associated ARDS. We analyzed mtDNA data from 687 peripheral whole-blood samples within 24 h of sepsis diagnosis from unrelated Spanish patients with sepsis (264 with ARDS) included in the GEN-SEP study. The wb-mtDNA copies were obtained from the array intensities of selected probes, with 100% identity with mtDNA and with the largest number of mismatches with the nuclear sequences, and normalized across the individual-probe intensities. We used Cox regression models for testing the association with 28-day survival. We observed that wb-mtDNA copies were significantly associated with 28-day survival in ARDS patients (hazard ratio = 3.65, 95% confidence interval = 1.39–9.59, p = 0.009) but not in non-ARDS patients. Our findings support that wb-mtDNA copies at sepsis diagnosis could be considered an early prognostic biomarker in sepsis-associated ARDS patients. Future studies will be needed to evaluate the mechanistic links of this observation with the pathogenesis of ARDS.

## Introduction

The acute respiratory distress syndrome (ARDS) is a lung inflammatory process that develops primarily as a response to respiratory or systemic-induced sepsis, which causes a disproportionate mortality burden in the adult intensive care unit (ICU) and has disabling consequences for years in surviving patients (1–3). ARDS occurs in 7 cases per 100,000 people per year, although the estimate varies widely among studies since the clinical diagnostic criteria are nonspecific. Its overall mortality rate remains high in most series, around 30%–40% (2–4). ARDS still has no effective and efficient treatment despite multiple studies that have focused on identifying the pathophysiology and improving the prognosis of these patients since it was first described. To date, lung-protective mechanical ventilation (MV) remains the main standard supportive ARDS treatment (5, 6) and there is no specific pharmacological therapy for it. Thus, identifying specific biomarkers will help to develop early therapeutic and preventive therapies, while assisting in predicting the prognosis of individual ARDS patients (3, 7, 8).

Mitochondria are a bioenergetic and biosynthetic cell organelle and a signaling hub that controls several important cellular functions, including cell survival and differentiation, as well as functioning of inflammatory responses (9, 10). The multiorgan and cellular dysfunction underlying sepsis and leading to ARDS could trigger mitochondrial dysfunction, which is characterized by fragmentation of the mitochondria and loss of integrity of mitochondrial DNA (mtDNA) (11). Previous studies have shown that, while the cellular mtDNA levels decrease, the cell-free mtDNA levels increase in response to a stimulus due to major trauma or a microbial infection (12–16). Based on evidence from animal models (17) and patient studies (18, 19), circulating mtDNA levels have been proposed as a potential biomarker for the systemic inflammatory response and lung injury after major trauma.

Cell-free mtDNA is considered a molecular pattern associated with damage (DAMPs) and could act as a critical activator of the innate immune system and inflammation (7, 9, 20, 21). Circulating cell-free mtDNA, measured by quantitative PCR (qPCR), has been associated with the overall 28-day mortality in ICU patients (22). Among ARDS patients, plasma mtDNA levels measured by qPCR on day 7 after diagnosis were significantly higher among non-surviving patients (23). Similarly, the plasma mtDNA levels among sepsis patients admitted to the emergency room were significantly higher among those who did not survive, and a score combining their levels with plasma lactate concentration considerably improved the 28-day mortality prediction (24). In fact, molecular patterns associated with mtDNA damage in transfusion products significantly contribute to the incidence of ARDS after massive transfusions (25).

Based on this evidence, and following a pragmatic approach, we tested the association of array-based measures of whole-blood mtDNA (wb-mtDNA) copies within 24 h of sepsis diagnosis with 28-day patient survival. We hypothesized that early wb-mtDNA measurements could be associated with mortality in patients with sepsis and ARDS.

## Methods

### Study Population

Peripheral blood samples and clinical information from 687 unrelated adult patients of European ancestry aged between 18 and 93 years from the network of Spanish postsurgical units and ICUs (GEN-SEP study) were used for this study (**Table 1**). The GEN-SEP cohort is a national, multicenter, observational study conducted in Spain between January 2002 and June 2019. For the purpose of this study, sepsis was defined according to the Third International Consensus Definitions for Sepsis (26). ARDS was defined according to the Berlin definition criteria (1). All participants gave written informed consent, and the study was approved by the Research Ethics Committee from all participating centers.

**Table 1:** Demographic and clinical features among sepsis, non-ARDS, and ARDS related to sepsis cases from the GEN-SEP study.

	All sepsis (N = 687)	Non-ARDS (N = 423)	ARDS (N = 264)	p-value*
Gender, % male (N)	63 (430)	60 (255)	66 (175)	0.133
Age, mean years ± SD	64 ± 15	64 ± 15	63 ± 14	0.139
BMI, mean ± SD	27 ± 6	27 ± 5	29 ± 7	0.029
SAPS, mean ± SD	47 ± 15	46 ± 15	49 ± 14	0.071
APACHE II, mean ± SD	20 ± 7	19 ± 7	22 ± 7	<0.001
Comorbidities^$^, % (N)	43 (256)	45 (174)	39 (82)	0.237
28-day mortality, % (N)	26 (181)	20 (85)	36 (96)	<0.001
ICU mortality, % (N)	28 (194)	19 (82)	42 (112)	<0.001
Days in hospital, mean ± SD	35 ± 40	33 ± 44	38 ± 33	<0.001
Days in ICU, mean ± SD	16 ± 23	12 ± 22	22 ± 23	<0.001
Organ dysfunction, % (N)				
Cardiovascular	90 (619)	87 (375)	92 (244)	0.137
Neurological systems	22 (152)	20 (85)	25 (67)	0.128
Coagulation	24 (168)	23 (98)	26 (70)	0.386
Hepatic	17 (117)	18 (75)	16 (42)	0.579
Renal	37 (252)	35 (150)	39 (102)	0.476
Respiratory	59 (404)	37 (157)	94 (247)	<0.001
Total SOFA^#^, mean ± SD	8 ± 4	8 ± 4	8 ± 4	0.257
Partial pressure of oxygen (PaO_2_), mean ± SD	109 ± 47	116 ± 51	96 ± 37	<0.001
Sepsis of pulmonary origin, % (N)	34 (229)	25 (103)	48 (126)	<0.001
Pathogen, % (N)				
Gram-positive	26 (126)	24 (74)	30 (52)	0.151
Gram-negative	35 (171)	35 (108)	36 (63)	0.747
Others^+^	29 (139)	29 (93)	26 (46)	0.532

*p-value calculated between non-ARDS and ARDS patients. Comparisons for gender, comorbidities, 28-day mortality, ICU mortality, sepsis of pulmonary origin, organ dysfunction, and pathogen were conducted by a chi-square test. The rest of variables were compared using the Mann–Whitney U-test.

^$^Includes: cancer, age >80 years, hepatopathy, valvular disease, immunodeficiency, severe brain damage, morbid obesity, chronic disease, autoimmune disease, pregnancy, myopathy, pneumonia, and serious recurrent infections.

^#^Total SOFA: sum of the cardiovascular, neurological systems, coagulation, hepatic, renal, and respiratory SOFA scores.

^+^Includes: mixed Gram-positive and Gram-negative infection, fungi, virus, and polymicrobial.

APACHE II, Acute Physiology and Chronic Health Evaluation II; BMI, body mass index; ICU, intensive care unit; SAPS, Simplified Acute Physiology Score II; SOFA, Sequential Organ Failure Assessment.

### Measures of Whole-Blood mtDNA Levels

DNA was purified using a commercial column-based solution (Illustra™ blood genomicPrep Mini Spin Kit) from peripheral blood drawn within 24 h of sepsis diagnosis, and the concentration was measured on the Qubit 3.0 fluorometer with the dsDNA HS Assay kit (Thermo Fisher Scientific). All samples were assessed for single-nucleotide polymorphisms (SNPs) across the genome using the Axiom Genome-Wide Human CEU 1 Array data (Thermo Fisher Scientific) in the National Genotyping Center (CeGen), Universidad de Santiago de Compostela Node, Spain. The intensity data were processed using AffyPipe v2.10.0 (27), following the quality controls recommended by the manufacturer. Further genotyping quality controls were performed with the R environment v3.6.0 and PLINK v1.07 (28). Samples with genotype call rates <95% or with evidence of relatedness (PIHAT > 0.2) were removed from the study. Likewise, we excluded SNPs based on genotyping rate <95%, minor allele frequency (MAF) <0.01, and largely deviating from Hardy–Weinberg expectations (p <1×10^-6^). Principal components (PCs) to assess genetic heterogeneity among patients were obtained from a subset of approximately 100,000 independent variants using PLINK v1.90 (29), and the first 5 PCs were used for sensitivity analysis.

To obtain a measure of the wb-mtDNA copies, we selected mtDNA probes from the array data and normalized (log R ratio) across the individual-probe intensities of the cohort following the methodology described by Tin and colleagues (11). To ensure specificity of the estimations, we used the average of the GC content-corrected intensities of the array probes targeting the human mtDNA with 100% identity, with the largest number of mismatches against the nuclear sequences based on BLAST (https://blast.ncbi.nlm.nih.gov/Blast.cgi). Probes behaving as outliers (beyond 1.5 SD of the mean) for the corrected intensities were then removed from the analysis. These filtering steps left us with a total of 17 probes to obtain the wb-mtDNA copy estimates, which were finally available for a total of 687 sepsis patients, where 181 subjects died in the ICUs within 28 days from sepsis onset. A total of 264 patients developed ARDS, and 96 of them died within 28 days from sepsis onset (**Table 1**).

### Statistical Analyses

The statistical power of the study was estimated using the formula n_event_ = (4*(Z_alfa_+Z_beta_)^2^)/[Ln(RR)]^2^ (30), supporting that as few as 88 events were needed to reach 90% statistical power. The “survival” R package 3.1-12 (31) was used to model the association between wb-mtDNA copies and 28-day survival in all patients with sepsis (N = 687), in those who developed ARDS (N = 264), and in non-ARDS patients (N = 423). Cox regressions and Kaplan–Meier analyses were performed. Sensitivity analyses were used to evaluate the effects of demographic and clinical variables on the Cox regression models. Finally, receiver operating characteristic (ROC) curves and their area under the curve (AUC) estimates were assessed with “pROC” R package 1.17.0.1 (32).

## Results

Demographic and clinical features of all sepsis patients and of those with or without ARDS are shown in **Table 1**. As expected, there were large and significant differences between ARDS and non-ARDS patients for their severity scores, hospital and ICU length of stay, mortality rate, and physiological variables such as the partial pressure of oxygen (PaO_2_). There were differences in the organ dysfunction among ARDS and non-ARDS patients (ANOVA, p < 0.001). However, as expected, they were due to the lung affectation. On the other hand, for the pathogens, we did not observe significant differences between ARDS and non-ARDS patients (ANOVA, p = 0.127).

We first found that the association between wb-mtDNA copies within 24 h of sepsis diagnosis and 28-day survival among all patients with sepsis from GEN-SEP was significant (**Table 2**). We then tested the same model stratifying the sepsis patients by those who did not develop ARDS and those who developed ARDS. In all models, the proportional risk assumption was held. Although we did not observe any association between wb-mtDNA copies and 28-day survival in septic non-ARDS patients, we found a strong association in septic patients who developed ARDS (hazard ratio [HR] = 3.65, 95% confidence interval [CI] 1.39–9.59, p = 0.009). Taken together, this indicates that the significant association between the wb-mtDNA copies within 24 h of sepsis diagnosis and 28-day survival observed among all GEN-SEP patients could be explained by those developing ARDS. A Kaplan–Meier analysis of the 28-day mortality among the ARDS patients reinforced this observation (log-rank test p = 0.037) (**Supplementary Figure S1** **)**.

**Table 2:** Association results of wb-mtDNA copy number with 28-day mortality.

Cohorts (N/events)	Hazard ratio (95% CI)	p-value
All patients (687/181)	2.39 (1.18–4.84)	0.015
Non-ARDS (423/85)	1.24 (0.44–3.51)	0.683
ARDS (264/96)	3.65 (1.39–9.59)	0.009

Given that there were demographic and clinical differences between ARDS patients that survived and that did not survive (**Table 3**), sensitivity analyses were conducted to ensure that those differences did not explain the association with wb-mtDNA. Note that there were no overall differences among ARDS patients in the organ dysfunction (ANOVA, p = 0.159) or in the pathogens (ANOVA, p = 0.985), therefore not affecting the sensitivity analyses. We found that the association was robust to model adjustments by variables that were not significantly different between survivor and non-survivor ARDS patients (e.g., gender, comorbidities, and the first five PCs of genetic heterogeneity). Likewise, the results of the univariate model with wb-mtDNA levels were similar to those with independent adjustment by age, SAPS, APACHE II score, and organ dysfunction, where the assumption of proportionality risk was held (**Table 4**). The only two adjusted models that did not hold for the proportionality risk assumption were those including as covariates the length of stay in ICU or in the hospital, for which conclusions should be taken with caution. Based on these findings, we tested multivariate models in the sensitivity analyses except those with a high proportion of missing data (i.e., SAPS) or were variable adjustments that turned into violated assumptions of the proportionality of risks in the sensitivity analyses (i.e., length of stay in ICU or in the hospital) (**Table 4**). We found that when including age and APACHE II score, the association of wb-mtDNA with survival was similar (HR = 4.51, 95%CI = 1.62–12.47, p = 0.004) and the proportionality of risk was held. The results were similar, and the proportionality of risk was also held, when the model adjusting for age and APACHE II also included the organ dysfunction categories that reached nominal significance in the differences between ARDS patients that survived and that did not survive (HR = 4.40, 95%CI = 1.55–12.53, p = 0.006).

**Table 3:** Demographic and clinical features of the ARDS patients.

	Survivors (N = 168)	Non-survivors (N = 96)	p-value^*^
Gender, % male (N)	66 (111)	67 (64)	1.000
Age, mean years ± SD	62 ± 14	66 ± 13	0.021
BMI, mean ± SD	29 ± 7	27 ± 5	0.192
SAPS, mean ± SD	47 ± 13	54 ± 16	0.004
APACHE II score, mean ± SD	21 ± 7	24 ± 7	<0.001
Comorbidities^$^, % (N)	40 (59)	37 (23)	0.707
Days in hospital, mean ± SD	51 ± 35	17 ± 11	<0.001
Days in ICU, mean ± SD	29 ± 26	11 ± 7	<0.001
Organ dysfunction, % (N)			
Cardiovascular	92 (155)	93 (89)	1.000
Neurological systems	21 (35)	34 (32)	0.031
Coagulation	21 (35	36 (35)	0.009
Hepatic	12 (20)	23 (22)	0.029
Renal	31 (52)	52 (50)	0.001
Respiratory	94 (158)	93 (89)	0.868
Total SOFA^#^, mean ± SD	8 ± 4	8 ± 5	0.834
Partial pressure of oxygen (PaO_2_), mean ± SD	95 ± 33	100 ± 44	0.627
Sepsis of pulmonary origin, % (N)	51 (84)	44 (42)	0.323
Pathogen, % (N)			
Gram-positive	29 (32)	34 (20)	0.537
Gram-negative	39 (45)	31 (18)	0.320
Others^+^	25 (28)	31 (18)	0.511

*p-value calculated between survivors and non-survivors. Comparisons for gender, comorbidities, sepsis of pulmonary origin, organ dysfunction, and pathogen were conducted by a chi-square test. The rest of variables were compared using the Mann–Whitney U-test.

^$^Includes: cancer, age >80 years, hepatopathy, valvular disease, immunodeficiency, severe brain damage, morbid obesity, chronic disease, autoimmune disease, pregnancy, myopathy, pneumonia, and serious recurrent infections.

^#^Total SOFA: sum of the cardiovascular, neurological systems, coagulation, hepatic, renal and respiratory SOFA scores.

^+^Includes: mixed Gram-positive and Gram-negative infection, fungi, virus and polymicrobial.

APACHE II, Acute Physiology and Chronic Health Evaluation II; BMI, body mass index; SAPS, Simplified Acute Physiology Score II; SOFA, Sequential Organ Failure Assessment.

**Table 4:** Association results of wb-mtDNA levels with 28-day survival in ARDS patients adjusting the models for the variables that were significantly different by mortality group.

	N	Hazard ratio (95% CI)	p-value
wb-mtDNA levels	264	3.65 (1.39–9.59)	0.009
Adjusted by age	264	3.99 (1.46–10.87)	0.007
Adjusted by SAPS	131	7.93 (1.57–39.93)	0.012
Adjusted by APACHE II score	258	4.26 (1.56–11.64)	0.005
Adjusted by organ dysfunction:			
Neurological systems	263	3.48 (1.30–9.27)	0.013
Coagulation	264	3.93 (1.47–10.42)	0.006
Hepatic	264	3.66 (1.39–9.62)	0.009
Renal	264	3.85 (1.46–10.20)	0.007
Adjusted by days in hospital*	228	5.77 (1.70–19.53)	0.005
Adjusted by days in ICU*	264	4.64 (1.67–12.87)	0.003
Adjusted by age, APACHE II	258	4.51 (1.62–12.57)	0.004
Adjusted by age, APACHE II, organ dysfunction	257	4.40 (1.55–12–53)	0.006

*Statistically significant for the Schoenfeld test of the proportionality of risks.

APACHE II, Acute Physiology and Chronic Health Evaluation II; ICU, intensive care unit; SAPS, Simplified Acute Physiology Score II.

Among the ARDS patients, the AUC of the wb-mtDNA copy number for the 28-day survival was 0.612 (95% CI = 0.541–0.683) (**Supplementary Figure S2**). However, this predictive value was similar to that provided by other clinical scores routinely used in clinical settings. As an example, for the same patients, the prognostic ability of the APACHE II score reached an AUC of 0.634 (95% CI = 0.565–0.704) (**Supplementary Figure S2**). Combining both the wb-mtDNA copy number and the APACHE II score in the models, the AUC slightly improved to 0.676 (95% CI = 0.609–0.742) although the AUC of the two curves (wb-mtDNA alone and wb-mtDNA plus APACHE II together) were not significantly different (p = 0.062) based on DeLong’s test for two correlated ROC curves.

## Discussion

Given the multifactorial risks involved in the prognostic trajectories of ICU patients (33), the identification of an ideal biomarker for predicting outcomes is a difficult task. One of the hallmarks of ARDS is the presence of inflammatory, protein-enriched pulmonary edema (1), caused by an increase in the permeability of the lung tissue (34, 35) and elevating the risk of death (2). It has been reported that DAMPs, including mtDNA, increase endothelial permeability through neutrophil-dependent and independent pathways (36). In a sufficiently powered cohort of Spanish patients recruited from a nationwide network of postsurgical ICUs, we describe evidence supporting that mtDNA copies measured in peripheral blood within 24 h of sepsis diagnosis were associated with 28-day survival in patients developing ARDS. Given that the association was absent among non-ARDS patients from the same series, our findings might suggest an ARDS-specific effect.

In agreement with our findings, Nakahira and colleagues observed an association between circulating cell-free mtDNA with overall patient 28-day mortality in the ICUs and if they combined mtDNA levels with other clinical parameters, the prediction of the ICU patients improved (22). Other studies have shown that high mtDNA plasma levels could be associated with sepsis and ARDS (19, 23, 24, 37). In one of these studies, high mtDNA plasma levels and a strong association with 28-day survival were observed in patients with sepsis and septic shock (24). Supporting our results, Huang and colleagues also observed a positive association between higher mtDNA plasma levels and 28-day mortality among patients with all-cause ARDS, although their findings revealed an association only with mtDNA measures of day 7 after diagnosis (23). Likewise, mtDNA plasma levels were also analyzed in other critically ill patients, such as the patients affected by the coronavirus disease 2019 (COVID-19) (38) or trauma patients (19), where also high mtDNA levels were associated with poor prognostics or outcomes of these diseases.

Among the strengths of this study, we recognize that it was based on a well-phenotyped and clinically characterized cohort of sepsis and ARDS patients. In addition, the wb-mtDNA estimates were obtained with a method that is not subject to the sample conservation problems linked to the qPCR (39). Related to this, given that we relied on an SNP array platform, we were able to perform model adjustments by the genetic heterogeneity, which is inherent in any heterogeneous patient population. Nevertheless, our study has some major limitations as well. The main weakness is that the models lacked adjustments by platelet count or the different cell populations, precluding determination of the contribution of the different types of white blood cells and platelets to the overall wb-mtDNA copy number estimation (13, 40). Another important limitation is that we were unable to adjust the models for other relevant clinical data that can be prognostic of ARDS such as creatinine, mean arterial pressure, Glasgow coma scale, and urine, among others. The analyses also lacked longitudinal measures that could have provided dynamical insights into mtDNA levels and outcomes (23). Besides, we have considered all the mtDNA content from the peripheral blood limiting the comparisons with other studies that have focused on the circulating cell-free fraction of mtDNA (22, 41). A final important limitation is that we were not able to infer causality.

## Conclusions

Wb-mtDNA copies measured within 24 h of sepsis diagnosis are significantly associated with 28-day survival in ARDS patients. Further studies should disentangle whether this association is independent of sepsis and whether the causality of wb-mtDNA elevation is involved in the pathogenesis of ARDS.

## Data Availability Statement

The raw intensity data supporting the conclusions of this article will be made available by the authors, without undue reservation.

## Ethics Statement

The studies involving human participants were reviewed and approved by the Ethics Committee for Drug Research from the Hospital Universitario de Canarias (Code: CHUNSC_2018-16). The patients/participants provided their written informed consent to participate in this study.

## Author Contributions

TH-B: data analysis, interpretation, and manuscript drafting. BG-G, HR-P, IM-R, JL-S, AC: performed the experiments, data analysis, and revision of the manuscript. MP-G, AR-P, DC, JB, AA, EG-H, NC, MG-G, EE, AM, DD, AG, JA, MS, JB, JG: performed the experiments, sample and clinical data collection, and data analysis. JV: analysis, interpretation, critical revision of the manuscript. CF: study conception and design, data analysis, interpretation, critical revision of the manuscript and conception of the project. All authors contributed to the article and approved the submitted version.

## Funding

This study was funded by the Instituto de Salud Carlos III, Madrid, Spain (CB06/06/1088, PI14/00844, PI16/00049, PI17/00610, PI20/00876, FI17/00177, FI18/00230, and CD19/00231), and by the European Regional Development Funds (ERDF) “A way of making Europe” from the European Union; by the agreement OA17/008 with Instituto Tecnológico y de Energías Renovables (ITER), Tenerife, Spain, to strengthen scientific and technological education, training, research, development, and innovation in Genomics, Personalized Medicine, and Biotechnology. The genotyping service was performed at CEGEN-PRB3-ISCIII, supported by grant PT17/0019 of the PE I+D+i 2013-2016, funded by the Instituto de Salud Carlos III and ERDF.

## Conflict of Interest

MG-G was employed by SPECTRUM, LLC.

The remaining authors declare that the research was conducted in the absence of any commercial or financial relationships that could be construed as a potential conflict of interest.

## Publisher’s Note

All claims expressed in this article are solely those of the authors and do not necessarily represent those of their affiliated organizations, or those of the publisher, the editors and the reviewers. Any product that may be evaluated in this article, or claim that may be made by its manufacturer, is not guaranteed or endorsed by the publisher.

## References

[1] Ranieri VM Rubenfeld GD Thompson BT Ferguson ND Caldwell E Fan E. Acute Respiratory Distress Syndrome: The Berlin Definition. J Am Med Assoc (2012) 307(23):2526–33. 10.1001/jama.2012.5669 22797452

[2] Bellani G Laffey JG Pham T Fan E Brochard L Esteban A. Epidemiology, Patterns of Care, and Mortality for Patients With Acute Respiratory Distress Syndrome in Intensive Care Units in 50 Countries. J Am Med Assoc (2016) 315(8):788–800. 10.1001/jama.2016.0291 26903337

[3] Matthay MA Zemans RL Zimmerman GA Arabi YM Beitler JR Mercat A. Acute Respiratory Distress Syndrome. Nat Rev Dis Prim (2018) 5(1):18. 10.1038/s41572-019-0069-0 PMC6709677 30872586

[4] Villar J Blanco J Kacmarek RM. Current Incidence and Outcome of the Acute Respiratory Distress Syndrome. Curr Opin Crit Care (2016) 22(1):1–6. 10.1097/MCC.0000000000000266 26645551

[5] Mauri T Lazzeri M Bellani G Zanella A Grasselli G. Respiratory Mechanics to Understand ARDS and Guide Mechanical Ventilation. Physiol Meas (2017) 38(12):R280–303. 10.1088/1361-6579/aa9052 28967868

[6] Shaw TD McAuley DF O’Kane CM. Emerging Drugs for Treating the Acute Respiratory Distress Syndrome. Expert Opin Emerg Drugs (2019) 24(1):29–41. 10.1080/14728214.2019.1591369 30841764

[7] Xu W Song Y. Biomarkers for Patients With Trauma Associated Acute Respiratory Distress Syndrome. Mil. Med Res (2017) 4(1):1–7. 10.1186/s40779-017-0134-5 28824814 PMC5558771

[8] García-Laorden I Lorente JA Flores C Slutsky AS Villar J. Biomarkers for the Acute Respiratory Distress Syndrome: How to Make the Diagnosis More Precise. Ann Transl Med (2017) 5(14):1–10. 10.21037/atm.2017.06.49 28828358 PMC5537109

[9] Krysko DV Agostinis P Krysko O Garg AD Bachert C Lambrecht BN. Emerging Role of Damage-Associated Molecular Patterns Derived From Mitochondria in Inflammation. Trends Immunol (2011) 32(4):157–64. 10.1016/j.it.2011.01.005 21334975

[10] Zhong FF Liang S Zhong Z. Emerging Role of Mitochondrial DNA as a Major Driver of Inflammation and Disease Progression. Trends Immunol (2019) 40(12):1120–33. 10.1016/j.it.2019.10.008 31744765

[11] Tin A Grams ME Ashar FN Lane JA Rosenberg AZ Grove ML. Association Between Mitochondrial DNA Copy Number in Peripheral Blood and Incident CKD in the Atherosclerosis Risk in Communities Study. J Am Soc Nephrol (2016) 27(8):2467–73. 10.1681/ASN.2015060661 PMC4978050 26794963

[12] Suliman HB Welty-Wolf KE Carraway MS Schwartz DA Hollingsworth JW Piantadosi CA. Toll-Like Receptor 4 Mediates Mitochondrial DNA Damage and Biogenic Responses After Heat-Inactivated E. Coli. FASEB J (2005) 19(11):1531–3. 10.1096/fj.04-3500fje 15994412

[13] Pyle A Burn DJ Gordon C Swan C Chinnery PF Baudouin SV. Fall in Circulating Mononuclear Cell Mitochondrial DNA Content in Human Sepsis. Intensive Care Med (2010) 36(6):956–62. 10.1007/s00134-010-1823-7 PMC4034433 20224905

[14] Zhang Q Raoof M Chen Y Sumi Y Sursal T Junger W. Circulating Mitochondrial Damps Cause Inflammatory Responses to Injury. Nature (2010) 464(7285):104–7. 10.1038/nature08780 PMC2843437 20203610

[15] Lu CH Chang WN Tsai NW Chuang YC Huang CR Wang HC. The Value of Serial Plasma Nuclear and Mitochondrial DNA Levels in Adult Community-Acquired Bacterial Meningitis. Qjm (2010) 103(3):169–75. 10.1093/qjmed/hcp201 20129945

[16] Cossarizza A Pinti M Nasi M Gibellini L Manzini S Roat E. Increased Plasma Levels of Extracellular Mitochondrial DNA During HIV Infection: A New Role for Mitochondrial Damage-Associated Molecular Patterns During Inflammation. Mitochondrion (2011) 11(5):750–5. 10.1016/j.mito.2011.06.005 21722755

[17] Gan L Zhong J Zhang R Sun T Li Q Chen X. The Immediate Intramedullary Nailing Surgery Increased the Mitochondrial DNA Release That Aggravated Systemic Inflammatory Response and Lung Injury Induced by Elderly Hip Fracture. Mediators Inflamm (2015) 2015:587378. 10.1155/2015/587378 26273137 PMC4530272

[18] Gu X Yao Y Wu G Lv T Luo L Song Y. The Plasma Mitochondrial DNA is an Independent Predictor for Post-Traumatic Systemic Inflammatory Response Syndrome. PloS One (2013) 8(8):1–8. 10.1371/journal.pone.0072834 PMC3748121 23977360

[19] Yamanouchi S Kudo D Yamada M Miyagawa N Furukawa H Kushimoto S. Plasma Mitochondrial DNA Levels in Patients With Trauma and Severe Sepsis: Time Course and the Association With Clinical Status. J Crit Care (2013) 28(6):1027–31. 10.1016/j.jcrc.2013.05.006 23787023

[20] Harrington JS Choi AMK and Nakahira K. Mitochondrial DNA in Sepsis. Curr Opin Crit Care (2017) 23(4):284–90. 10.1097/MCC.0000000000000427 PMC5675027 28562385

[21] Torralba D Baixauli F Villarroya-Beltri C Fernández-Delgado I Latorre-Pellicer A Acín-Pérez R. Priming of Dendritic Cells by DNA-Containing Extracellular Vesicles From Activated T Cells Through Antigen-Driven Contacts. Nat Commun (2018) 9(1):1–17. 10.1038/s41467-018-05077-9 29985392 PMC6037695

[22] Nakahira K Kyung SY Rogers AJ Gazourian L Youn S Massaro AF. Circulating Mitochondrial DNA in Patients in the ICU as a Marker of Mortality: Derivation and Validation. PloS Med (2013) 10(12):1–12. 10.1371/journal.pmed.1001577 PMC3876981 24391478

[23] Huang L Chang W Huang Y Xu X Yang Y Qiu H. Prognostic Value of Plasma Mitochondrial DNA in Acute Respiratory Distress Syndrome (ARDS): A Single-Center Observational Study. J Thorac Dis (2020) 12(4):1320–8. 10.21037/jtd.2020.02.49 PMC7212167 32395269

[24] Wang L W. Zhou W Wang K He S Chen Y. Predictive Value of Circulating Plasma Mitochondrial DNA for Sepsis in the Emergency Department: Observational Study Based on the Sepsis-3 Definition. BMC Emerg Med (2020) 20(1):1–7. 10.1186/s12873-020-00320-3 32299369 PMC7164211

[25] Simmons PJD Lee YL Pastukh VM Capley G Muscat CA Muscat DC. Potential Contribution of Mitochondrial (Mt) DNA Damage Associated Molecular Patterns (Damps) in Transfusion Products to the Development of Acute Respiratory Distress Syndrome (ARDS) After Multiple Transfusions. J Trauma Acute Care Surg (2017) 86(6):1023–9. 10.1097/TA.0000000000001421 PMC5472063 28301393

[26] Singer M Deutschman CS Seymour CW Shankar-Hari M Annane D Bauer M. The Third International Consensus Definitions for Sepsis and Septic Shock (Sepsis-3). J Am Med Assoc (2016) 315(8):801–10. 10.1001/jama.2016.0287 PMC4968574 26903338

[27] Nicolazzi EL Iamartino D Williams JL. Affypipe: An Open-Source Pipeline for Affymetrix Axiom Genotyping Workflow. Bioinformatics (2014) 30(21):3118–9. 10.1093/bioinformatics/btu486 PMC4609010 25028724

[28] Purcell S Neale B Todd-Brown K Thomas L Ferreira M Bender D. PLINK: A Tool Set for Whole-Genome Association and Population-Based Linkage Analyses. Am J Hum Genet (2007) 81(3):559–75. 10.1086/519795 PMC1950838 17701901

[29] Chang CC Chow CC Tellier L Vattikuti CAM Purcell S Lee JJ. Second-Generation PLINK: Rising to the Challenge of Larger and Richer Datasets. GigaScience (2015) 4(1):1–16. 10.1186/s13742-015-0047-8 25722852 PMC4342193

[30] Schoenfeld D. The Asymptotic Properties of Nonparametric Tests for Comparing Survival Distributions. Biometrika (1981) 68(1):316–9. 10.2307/2335833

[31] Therneau T. A Package for Survival Analysis in R. R Package Version 3.2-13. (2021). Available at: https://CRAN.R-project.org/package=survival.

[32] Robin X Turck N Hainard A Tiberti N Lisacek F Sanchez J-C. Proc: An Open-Source Package for R and s+ to Analyze and Compare ROC Curves. BMC Bioinf (2011) 12(1):77. 10.1186/1471-2105-12-77 PMC3068975 21414208

[33] Lin MT Albertson TE. Genomic Polymorphisms in Sepsis. Crit Care Med (2004) 32(2):569–79. 10.1097/01.CCM.0000110878.49476.42 14758181

[34] Barratt S Medford AR Millar AB. Vascular Endothelial Growth Factor in Acute Lung Injury and Acute Respiratory Distress Syndrome. Respiration (2014) 87(4):329–42. 10.1159/000356034 24356493

[35] Ourradi K Blythe T Jarrett C Barratt SL Welsh GI Millar AB. VEGF Isoforms Have Differential Effects on Permeability of Human Pulmonary Microvascular Endothelial Cells. Respir Res (2017) 18(1):1–12. 10.1186/s12931-017-0602-1 28578669 PMC5457598

[36] Sun S Sursal T Adibnia Y Zhao C Zheng Y Li H. Mitochondrial Damps Increase Endothelial Permeability Through Neutrophil Dependent and Independent Pathways. PloS One (2013) 8(3):e59989. 10.1371/journal.pone.0059989 23527291 PMC3603956

[37] Faust HE Reilly JP Anderson BJ Ittner CAG Forker CM Zhang P. Plasma Mitochondrial DNA Levels are Associated With ARDS in Trauma and Sepsis Patients. Chest (2020) 157(1):67–76. 10.1016/j.chest.2019.09.028 31622590 PMC6965693

[38] Scozzi D Cano M Ma L Zhou D Zhu JH O'Halloran JA. Circulating Mitochondrial DNA Is an Early Indicator of Severe Illness and Mortality From COVID-19. JCI Insight (2021) 6(4):e143299. 10.1101/2020.07.30.227553 PMC7934921 33444289

[39] Ruiz-Villalba A van Pelt-Verkuil E Gunst QD Ruijter JM van den Hoff MJB. Amplification of Nonspecific Products in Quantitative Polymerase Chain Reactions (Qpcr). Biomol Detect Quantif (2017) 14:7–18. 10.1016/j.bdq.2017.10.001 29255685 PMC5727009

[40] Shim HB Arshad O Gadawska I Côté HCF Hsieh AYY. Platelet MtDNA Content and Leukocyte Count Influence Whole Blood MtDNA Content. Mitochondrion (2020) 52:108–14. 10.1016/j.mito.2020.03.001 32156645

[41] Johansson PI Nakahira K Rogers AJ McGeachie MJ Baron RM Fredenburgh LE. Plasma Mitochondrial DNA and Metabolomic Alterations in Severe Critical Illness. Crit Care (2018) 22(1):1–9. 10.1186/s13054-018-2275-7 30594224 PMC6310975

