# Supplementary material for: Whole-Blood Mitochondrial DNA Copies Are Associated With the Prognosis of Acute Respiratory Distress Syndrome After Sepsis
Source: Front Immunol. 2021 Sep 7;12:737369. doi: 10.3389/fimmu.2021.737369 (PMC8453061; doi:10.3389/fimmu.2021.737369)
Supplement: Supplementary file 1 [file DataSheet_1.docx]

Supplementary Material

**Supplementary Figures**


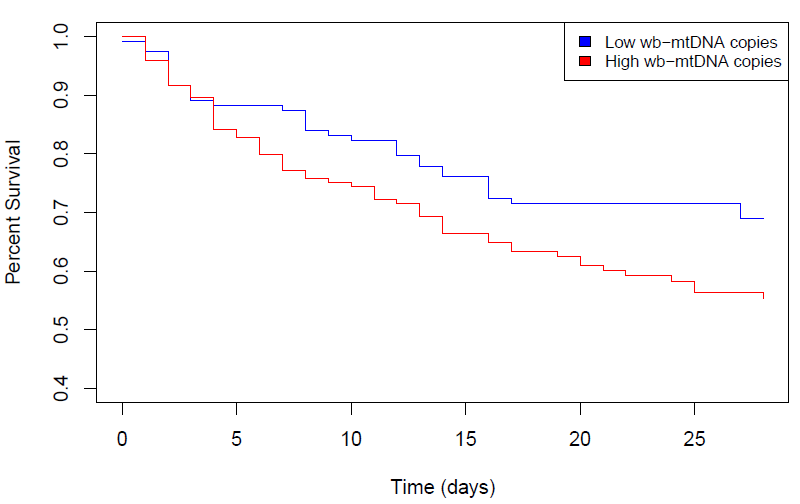


Figure S1. Kaplan-Meier analysis of 28-day survival of the ARDS patients by the wb-mtDNA copies. The red curve reflects the patients with the higher normalized intensity of wb-mtDNA with respect to the median while the blue curve reflects those with lower wb-mtDNA copies with respect to the median. The patients with the higher wb-mtDNA copies had lower survival rates (Logrank test p=0.037).


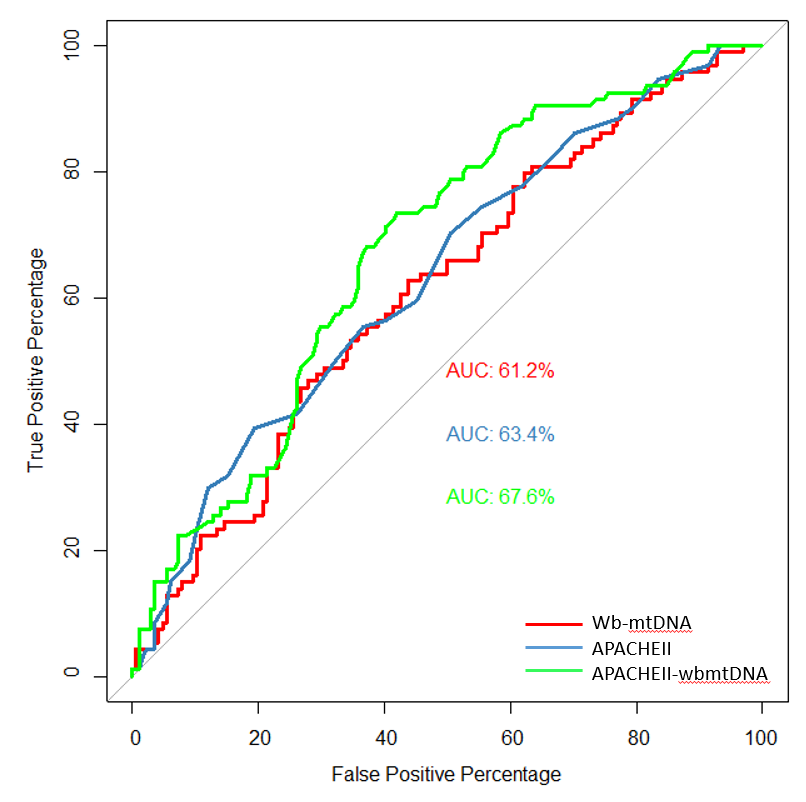


Figure S2. Receiver Operating Characteristic curves and the Area Under the Curve (AUC) estimates of the wb-mtDNA copies and APACHE II score at inclusion, by separate and combined, as predictors of 28-day mortality in ARDS patients.
